# Haloperidol affects bones while clozapine alters metabolic parameters - sex specific effects in rats perinatally treated with phencyclidine

**DOI:** 10.1186/s40360-017-0171-4

**Published:** 2017-10-11

**Authors:** Tatjana Nikolić, Milan Petronijević, Jelena Sopta, Milica Velimirović, Tihomir Stojković, Gordana Jevtić Dožudić, Milan Aksić, Nevena V. Radonjić, Nataša Petronijević

**Affiliations:** 1 Institute of Medical and Clinical Biochemistry, School of Medicine, University of Belgrade, Belgrade, Serbia; 2 Military Medical Academy, Clinic of Rheumatology, University of Defence, Belgrade, Serbia; 3 Institute of Pathology, School of Medicine, University of Belgrade, Belgrade, Serbia; 4 Institute of Anatomy “Niko Miljanic”, School of Medicine, University of Belgrade, Belgrade, Serbia; 5 Department of Psychiatry, University of Connecticut School of Medicine, Farmington, CT USA

**Keywords:** Schizophrenia, Bone mineral density, Haloperidol, Clozapine, Corticosterone, Phencyclidine, Interleukin

## Abstract

**Background:**

The presentation of schizophrenia (SCH) symptoms differs between the sexes. Long-term treatment with antipsychotics is frequently associated with decreased bone mineral density, increased fracture risk and metabolic side effects. Perinatal phencyclidine (PCP) administration to rodents represents an animal model of SCH. The aim of this study was to assess the effects of chronic haloperidol and clozapine treatment on bone mass, body composition, corticosterone, IL-6 and TNF-α concentrations and metabolic parameters in male and female rats perinatally treated with PCP.

**Methods:**

Six groups of male and six groups of female rats (*n* = 6-12 per group) were subcutaneously treated on 2nd, 6th, 9th and 12th postnatal day (PN), with either PCP (10 mg/kg) or saline. At PN35, one NaCl and PCP group (NaCl-H and PCP-H) started receiving haloperidol (1 mg/kg/day) and one NaCl and PCP group (NaCl-C and PCP-C) started receiving clozapine (20 mg/kg/day) dissolved in drinking water. The remaining NaCl and PCP groups received water. Dual X-ray absorptiometry measurements were performed on PN60 and PN98. Animals were sacrificed on PN100. Femur was analysed by light microscopy. Concentrations of corticosterone, TNF-α and IL-6 were measured in serum samples using enzyme-linked immunosorbent assay (ELISA) commercially available kits. Glucose, cholesterol and triacylglycerol concentrations were measured in serum spectrophotometrically.

**Results:**

Our results showed that perinatal PCP administration causes a significant decrease in bone mass and deterioration in bone quality in male and female rats. Haloperidol had deleterious, while clozapine had protective effect on bones. The effects of haloperidol on bones were more pronounced in male rats. It seems that the observed changes are not the consequence of the alterations of corticosterone, IL-6 and TNF-α concentration since no change of these factors was observed. Clozapine induced increase of body weight and retroperitoneal fat in male rats regardless of perinatal treatment. Furthermore, clozapine treatment caused sex specific increase in pro-inflammatory cytokines.

**Conclusion:**

Taken together our findings confirm that antipsychotics have complex influence on bone and metabolism. Evaluation of potential markers for individual risk of antipsychotics induced adverse effects could be valuable for improvement of therapy of this life-long lasting disease.

**Electronic supplementary material:**

The online version of this article (10.1186/s40360-017-0171-4) contains supplementary material, which is available to authorized users.

## Background

Schizophrenia (SCH) is a severe neuropsychiatric illness characterized by presence of positive (hallucinations, delusions, thought disorders) and negative (flattened affect, social withdrawal, attention deficit) symptoms and cognitive dysfunction (problems with attention, working memory and executive functions) [1–3].

Long-term treatment with “typical” antipsychotics such as haloperidol, or with “atypical” antipsychotics such as clozapine, is often required for treating the symptoms. Typical antipsychotics, that primarily inhibit dopaminergic pathways, are more likely to produce extrapyramidal side effects due to dopamine blockade in the basal ganglia. “Atypical” antipsychotics have both anti-dopaminergic and anti-serotonergic activity. These drugs have greater anti-psychotic efficiency and fewer extrapyramidal side effects [4]. However, treatment with antipsychotics, especially atypical is frequently associated with distinctive metabolic side effects including obesity [5], type 2 diabetes and dyslipidaemia that contribute to overall morbidity and mortality [6]. Decreased bone mineral density (BMD) and increased fracture risk are noticed in SCH patients, who receive long-term antipsychotic therapy [7]. Changes of bone mass [8] and metabolic parameters [9] are also found in drug naive or SCH patients minimally exposed to antipsychotics. It is not clear whether the disease per se or a life style (smoking, sedentary, nutrition, vitamin D deficiency, etc) together with antipsychotics is the cause of observed bone and metabolic changes.

Several studies reported that long-term use of antipsychotics may lead to a decrease in BMD and osteoporosis in relation to hyperprolactinemia [10–12] and currently, it is a generally accepted approach to categorize antipsychotics according to their effects on prolactin levels as prolactin-raising (PR) and prolactin-sparing (PS) antipsychotics [13–15]. However, recent meta-analysis of Stubbs et al. [16] has included nineteen studies and found that the hyperprolactinemia and taking of PR, as well as smoking, duration of illness and body mass index (BMI) were not related to the prevalence of osteoporosis. Significant correlation was seen with older age and a higher percentage of males indicating that men suffering from SCH are more vulnerable to osteoporosis and osteopenia than women. Recent review of Chen et al. [17] has also pointed out the gender differences in the effects of antipsychotics on BMD in patients with schizophrenia and has suggested that gender specific risk factors should be considered for intervention on bone loss. The authors have also found the evidences from previous studies, regarding the effects of antipsychotic-related hyperprolactinemia on BMD, inconclusive mainly due to cross-sectional study design or lack of adequate control groups.

Beside antipsychotic induced hyperprolactinemia and life style, several other factors including increased activity of some interleukins and hypercortisolaemia could be responsible for accelerated osteoporosis seen in SCH patients [18]. Elevated levels of cortisol, the end hormone produced by the activation of the hypothalamic–pituitary–adrenal (HPA) axis, and of inflammatory markers, such interleukin (IL)-6, have been consistently shown in first-episode, drug-naive schizophrenia patients [19–21]. Also, evidences suggest that the levels of both cortisol and interleukins are affected by antipsychotic medications [22, 23].

Attempts to elucidate the causal mechanisms, identify psychosis-specific biomarkers of SCH, and to understand the effects of drugs, have led to the development of animal models [24]. One of the actual pharmacological animal models of this disease is perinatal phencyclidine (PCP) administration to rodents [25, 26].

PCP is a non-competitive antagonist of glutamatergic N-methyl-D-aspartate (NMDA) receptors, capable to produce a broad spectrum of effects in healthy human volunteers that resemble SCH. PCP may induce positive symptoms including agitation, audiovisual hallucinations and paranoid delusions, negative symptoms represented as blunting of affect and apathy, as well as, cognitive disorders [27, 28].

Schizophrenia like symptoms have been repeatedly found in rats perinatally treated with phencyclidine including deficit in the prepulse inhibition of acoustic startle response, a measure used in the assessment of sensorimotor gating deficits in schizophrenia, increased locomotor activity that is accepted as an index of positive symptoms and disturbances in working memory that are a core feature of the cognitive dysfunction [26, 29–33]. Disturbance of baseline temperature [34] as well as decreased glutathione levels and altered antioxidant defence [25, 35], decreased number of several classes of interneurons [36] and alterations of mitochondria and apoptosis and autophagy processes [37] have also been reported.

Although, there are several studies describing the effects of antipsychotics on bone [38–40] and metabolic parameters [41–43] in animals, there is a lack of investigations concerning effects of antipsychotics on these parameters in experimental animal model of SCH. Since our recent investigation has revealed reduced bone mass in male rats in PCP animal model of SCH and protective effect of atypical antipsychotic risperidone [44], the aim of this study was to assess the effects of chronic haloperidol (belonging to PR antipsychotics) and clozapine (belonging to PS antipsychotics) treatment on bone mass, body composition, corticosterone, IL-6 and TNF-α concentrations and metabolic parameters in male and female rats perinatally treated with PCP.

## Methods

### Animals

Twelve timed pregnant Wistar rats were obtained at day 14 of pregnancy. The animals were housed individually in wire-hanging cages located within a temperature-controlled animal vivarium maintained under a 12:12-h light/dark schedule (lights on at 07:00 h). Food and water were available ad libitum throughout the experiment. All experiments were carried out according to the Directive of the European Parliament and of the Council (2010/63/EU) and approved by The Ethical Committee of the University of Belgrade and Ministry of Agriculture and Environmental Protection Serbia (Permission No 323-07-09403/2015-05/2).

Within 12 h of parturition, the pups from dams were cross fostered and then randomly assigned to one of lactating dams. Day of birth was considered to be postnatal (PN) day 0. The animals were treated on 2nd, 6th, 9th and 12th PN days, with either phencyclidine (PCP, six groups) or saline (NaCl 0.9%, six groups). PCP (Sigma, St. Louis, MO) was dissolved in a vehicle solution of 0.9% physiological saline (0.001 g/ml) and injected subcutaneously (s.c.) in the interscapular region (10 mg/kg). The dose and time course of the treatment were selected according to published studies [25, 26, 34–37, 44–46]. The vehicle control was saline alone injected s.c. in the same volume as PCP. The pups were kept in the same litters and weaned at PN day 30, when they were separated from the dams and classified according to the sex. In the present study both male and female rats were included.

### Treatment groups

Six groups of male and female rats were studied:

1) NaCl group (control): animals were perinatally treated with NaCl (11 males and 6 females); from PN 35 acetic acid (final concentration 1 mM) was added to drinking water in an equivalent concentration as it was used for dissolving of antipsychotics.

2) PCP group: animals were perinatally treated with PCP (7 males and 6 females); from PN 35 acetic acid was added to drinking water as in group 1.

3) NaCl–H group: animals perinatally treated with NaCl (6 males and 10 females); from PN 35 have started to receive haloperidol (H) (Krka, Slovenia) at dose 1 mg/kg/day.

4) PCP–H group: animals perinatally treated with PCP (7 males and 13 females); from PN 35 received haloperidol therapy in the same way as group 3.

5) NaCl–C group: animals perinatally treated with NaCl (10 males and 6 females); from PN 35 have started to receive clozapine (C) (Sandoz, Germany) at dose 20 mg/kg/day.

6) PCP–C group: animals perinatally treated with PCP (6 males and 12 females); from PN 35 received clozapine therapy in the same way as group 5.

Haloperidol and clozapine dosage were based on molecular, in vitro and in vivo, occupancy studies in adult animals [47, 48]. Drug dosing was based on the weight of the animals and the average daily fluid consumption. The drugs were administered orally in drinking water until PN 100. The antipsychotics were dissolved in 0.1 M acetic acid and subsequently diluted (1:100) for daily drug administration in drinking water [49, 50].

### Bone density and body composition measurements by DXA

Dual X-ray absorptiometry (DXA) scans were performed at PN day 60 and 98 to measure areal BMD, bone mineral content (BMC), fat and lean using the Lunar Prodigy Advance DXA (GE Healthcare Lunar Corp., Madison, USA) with software EnCore2007 and option Small animal body analysis. Anaesthesia consisted on an intra-peritoneal injection of a solution of thiopental sodium (40 mg/kg bodyweight, Sigma, St. Louis,MO). Measurements were obtained in standard mode, area 20 × 30 cm, voltage 76.0 kV, dose 1.9 μGy. BMD (mg/cm^2^) and BMC (mg/cm) were measured in regions of interest (femur, spine and total body which besides spine and femur have included head, trunk and ribs). Fat and lean tissue quantities (g) were presented as legs, trunk and total content.

### Tissue collection

Body weight was measured at PN 100 and animals were sacrificed by cervical dislocation and decapitation without anaesthesia. Rats were fasted for 12 h before sacrifice. Blood samples were collected and serum was obtained by centrifugation (15 min, 3000 rpm). Serum samples were stored at −80 °C. Retroperitoneal and periepididymal adipose tissues were isolated and weighed. The femur was dissected and further processed for light microscopic pathology analysis.

### Determination of IL-6, TNF-α and corticosterone concentration

Concentrations of corticosterone, TNF-α and IL-6 were measured in serum samples using enzyme-linked immunosorbent assay (ELISA) commercially available kits. Concentrations of corticosterone and TNF-α were measured in serum samples (dilution applied was 1:10 and 1:2 respectively), using Corticosterone EIA Kit, IDS and Rat TNF-α ELISA Kit, Invitrogen. Concentrations of IL-6 were measured in non-diluted serum samples, using Rat IL-6 ELISA Kit, Novex, Life technologies, following the manufacturer’s instructions. Absorbance was read at 450 nm using a microplate reader. The results were calculated in comparison with the standard curve. Each sample was run in duplicate and the average was calculated.

For the corticosterone ELISA Kit the calculated intra-assay % CV was 4.9 for the concentration of 4.6 ng/ml and 3.8 for the concentration of 45.7 ng/ml and the calculated inter-assay % CV was 7.8 for the concentration of 4.7 ng/ml and 7.7 for the concentration of 45.2 ng/ml. For the TNF-α ELISA Kit the calculated intra-assay % CV was 6.9 for the concentration of 130.7 pg/ml and 4.3 for the concentration of 912.1 pg/ml while inter-assay % CV was 9.0 for the concentration of 135 pg/ml and 7.8 for the concentration of 970.9 pg/ml. For the IL-6 ELISA Kit the calculated intra-assay % CV was 5.8 for the concentration of 49.2 pg/ml and 3.5 for the concentration of 845.6 pg/ml while the inter-assay % CV was 8.8 for the concentration of 51.6 pg/ml and 6.3 for the concentration of 865.8 pg/ml.

### Spectrophotometric measurement of glucose, cholesterol, triacylglycerol, calcium and phosphate concentration

Concentrations of glucose, cholesterol and triacylglycerol were measured spectrophotometrically using a commercially available enzymes assay kits (*GL 364, Randox, Crumlin, UK,* for glucose*; CH 200, Randox, Crumlin, UK* for cholesterol and *TR 1697*, *Randox, Crumlin, UK* for triacylglycerol). Concentrations of calcium and phosphate in serum were measured spectrophotometrically (using Arsenazo III method for calcium and UV for phosphate) with authomatic biochemical analyzer (Biosystem A15; Biosystems, Spain).

### Light microscopic analysis of femoral metaphysis

Analysis was performed by an investigator, blinded to the treatments. For tissue collection, the leg was disarticulated at the hip. For microscopic histological evaluation, femurs were removed and immediately fixed in 10% neutral-buffered formalin. The femur was cleaned of soft tissue, placed in decalcifying solution (8% HCl from 37% (*v*/v) concentrate and 10% formic acid from 89% (v/v) concentrate in PBS) for ∼24 h at 37 °C, dehydrated in graded ethanol, and then embedded in paraffin. Three 5-μm-thick paraffin-embedded horizontal bone sections were cut from the proximal end of the diaphysis, dyed with a haematoxylin–eosin, von Kossa and Masson’s trichrome stains and analysed by light microscopy and microscope Nikon Eclipse Ci with control unit DS-L3 and pre-installed software.

### Statistical analysis

All results are presented as mean values with standard error of the mean (SEM) and were analysed using the one way ANOVA with Fisher’s post hoc test. *P* value less than 0.05 was considered statistically significant.

## Results

### Bone density and body composition measurements by DXA

#### Changes of BMD were more pronounced in male rats treated with PCP and/or antipsychotics

**Fig. 1:**
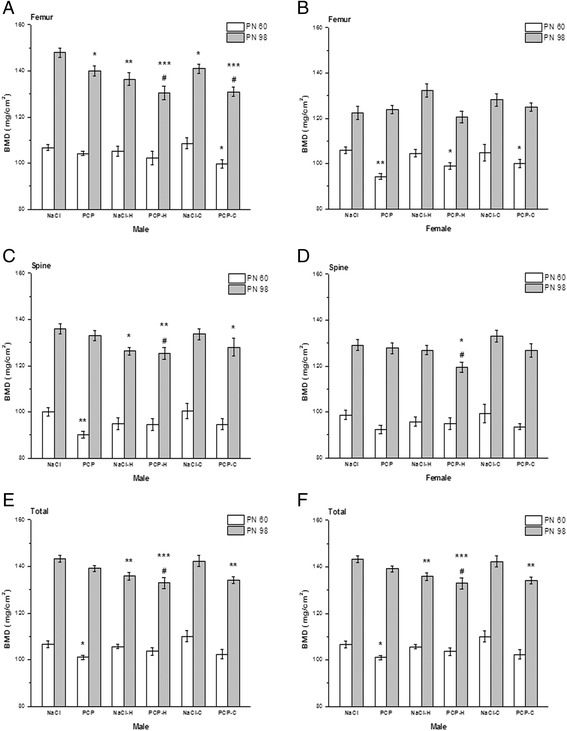
Effects of perinatal phencyclidine (PCP) treatment, haloperidol (H) and clozapine (C) on bone mineral density (BMD) on postnatal (PN) day 60 and PN 98 in male (**a**, **c** and **e**) and female (**b**, **d** and **f**) rats. Results are presented as mean values with standard error of the mean (SEM). ^*^ *p* < 0.05; ^**^ *p* < 0.01;^***^ *p* < 0.001 - comparing to control group. ^#^ *p* < 0.05– comparing to PCP group

Effects of perinatal PCP treatment, haloperidol and clozapine on BMD are presented in Fig. 1.

Changes in bone mineral density were more prominent on PN 98 in male rats (Fig. 1a–e). ANOVA showed a significant changes of femur [F(5,41) = 7.06, *p* < 0.001], spine [F(5,41) = 2.84, *p* < 0.05] and total [F(5,41) = 5.26, *p* < 0.001] BMD between investigated groups. Post hoc analysis with Fisher’s test showed significantly lower femur BMD in PCP (*p* < 0.05), NaCl-H (*p* < 0.01), NaCl-C group (*p* < 0.05) and PCP-H and PCP-C group (*p* < 0.001) compared to controls. Spine BMD was significantly lower in NaCl-H and PCP-C group (*p* < 0.05), as well as, in PCP-H (*p* < 0.01) group while total BMD was reduced in NaCl-H and PCP-C group (*p* < 0.01) and PCP-H group (*p* < 0.001) compared to controls.

In female rats (Fig. 1b–f) the only observed change of BMD on PN 98 was a significant decrease of spine BMD [F(5,47) = 3.27, *p* < 0.01] in PCP-H (*p* < 0.05) compared to control and PCP animals.

#### Haloperidol unlike clozapine further promoted PCP induced changes in BMC in male rats

**Fig. 2:**
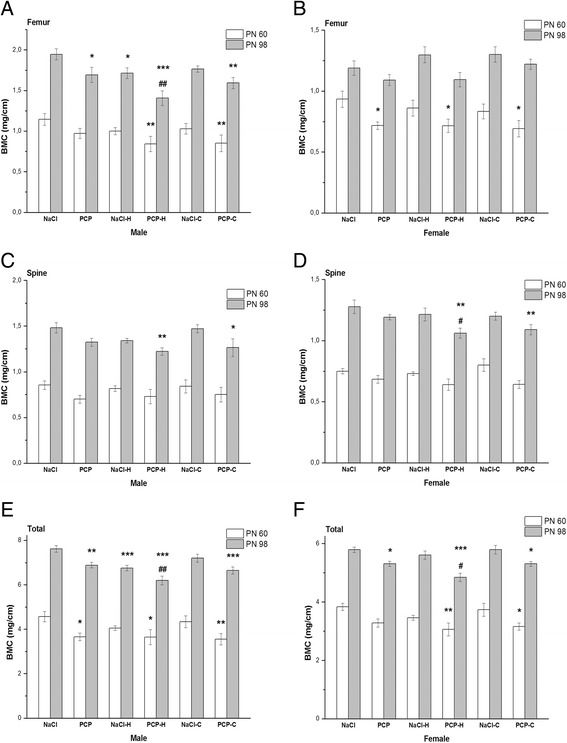
Effects of perinatal phencyclidine PCP treatment, haloperidol (H) and clozapine (C) on bone mineral content (BMC) on postnatal (PN) day 60 and PN 98 in male (**a**, **c** and **e**) and female (**b**, **d** and **f**) rats. Results are presented as mean values with standard error of the mean (SEM). ^*^ *p* < 0.05; ^**^ *p* < 0.01;^***^ *p* < 0.001 - comparing to control group. ^#^ *p* < 0.05; ^##^ *p* < 0.01– comparing to PCP group

Effects of perinatal PCP treatment, haloperidol and clozapine on bone mineral content are presented in Fig. 2.

Changes of BMC in treated groups were more prominent on PN 98. In male rats ANOVA showed a significant changes of femur [F(5,41) = 5.89, *p* < 0.001], spine [F(5,41) = 3.74, *p* < 0.01] and total [F(5,41) = 8.45, p < 0.001] BMC between investigated groups (Fig. 2a–e). Post hoc analysis showed significantly lower femur BMC in PCP and NaCl-H group (*p* < 0.05), PCP-H (*p* < 0.001) and PCP-C group (*p* < 0.01) compared to control. Spine BMC was significantly reduced in PCP-H (*p* < 0.01) and PCP-C group (*p* < 0.05) and total BMC in PCP (*p* < 0.01), NaCl-H (*p* < 0.001), PCP-H (*p* < 0.001) and PCP-C group (*p* < 0.001) compared to control.

On PN 98 in female rats, ANOVA showed a significant changes of spine [F(5,47) = 3.53, *p* < 0.01] and total [F(5,47) = 8.9, *p* < 0.001] BMC between investigated groups (Fig. 2b–f). Post hoc analysis with Fisher’s test showed significantly lower spine BMC in PCP-H and PCP-C group (*p* < 0.01) while total BMC was significantly reduced in PCP (*p* < 0.05), PCP-H (*p* < 0.001) and PCP-C group (*p* < 0.05) compared to control.

#### PCP and/or antipsychotics have different effects on fat and lean contents in male and female rats

Effects of perinatal PCP treatment, haloperidol and clozapine on fat content are presented in Additional file 1: Table S1. On PN 98 in male rats, ANOVA showed a significant changes of legs [F(5,41) = 4.37, *p* < 0.01] and total [F(5,41) = 4.25, *p* < 0.01], while there were no differences in trunk fat content [F(5,41) = 2.76, *p* > 0.05] between investigated groups. Post hoc analysis with Fisher’s test has shown significantly increased legs fat content in PCP (*p* < 0.01) and NaCl-C group (*p* < 0.05) compared to control.

On PN 98 in female rats, ANOVA showed a significant changes of trunk [F(5,47) = 7.15, *p* < 0.001] and total [F(5,47) = 5.70, *p* < 0.001], while there were no changes in legs fat content between evaluated groups [F(5,47) = 1.84, p > 0.05]. Post hoc analysis with Fisher’s test has shown significantly decreased trunk fat content in PCP (*p* < 0.001), NaCl-H (*p* < 0.05), PCP-H (*p* < 0.001) and PCP-C group (*p* < 0.01) and total fat content in PCP and PCP-H group (*p* < 0.001), as well as, in PCP-C group (*p* < 0.05) compared to control.

Effects of perinatal PCP treatment, haloperidol and clozapine on lean content are presented in Additional file 2: Table S2. On PN 98 in male rats ANOVA showed a significant changes of legs [F(5,41) = 8.1, *p* < 0.001], trunk [F(5,41) = 8.35, *p* < 0.001] and total lean content [F(5,41) = 11.73, *p* < 0.001]. Post hoc analysis with Fisher’s test has shown significantly increased legs lean in PCP (*p* < 0.05) and significantly reduced in PCP-H group (*p* < 0.01) compared to control. Trunk lean was significantly reduced in NaCl-H (*p* < 0.05), PCP-H (*p* < 0.001) and PCP-C (*p* < 0.05), while total lean was significantly reduced in PCP-H (*p* < 0.001) and PCP-C group (*p* < 0.05) compared to control.

On PN 98 in female rats, ANOVA showed a significant changes of legs [F(5,47) = 5.11, *p* < 0.001], trunk [F(5,47) = 5.72, *p* < 0.001] and total lean content [F(5,47) = 5.56, *p* < 0.001]. Post hoc analysis with Fisher’s test has shown significantly increased legs lean content in NaCl-H group (*p* < 0.01) and significant decrease of trunk (*p* < 0.001) and total (*p* < 0.01) lean content in PCP-H group compared to control.

### Light microscopic analysis of the femoral metaphysis

Light microscopic analysis of the femoral metaphysis is presented in Table 1. ANOVA showed significant changes of trabecular area in males [F(5,41) = 313.54, *p* < 0.001] and females [F(5,47) = 24,388.04, *p* < 0.001].

**Table 1:** Light microscopic pathology analysis of femur

		NaCl	PCP	NaCl-H	PCP-H	NaCl-C	PCP-C
Trabecular area (μm^2^)	M	25,825 ± 837	14,916 ± 689^***^	16,754 ± 1590^**^	5875 ± 1897^***##^	35,615 ± 2661^***^	35,624 ± 2755^***###^
	F	34,734 ± 1073	17,380 ± 714^***^	35,802 ± 2171	25,140 ± 1441^***##^	35,292 ± 1018	37,212 ± 977^***###^

Effects of perinatal phencyclidine (PCP) treatment, haloperidol (H) and clozapine (C) on trabecular area of the femoral metaphysis on PN 100 in male (M) and female (F) rats. Results are presented as mean values with standard error of the mean (SEM)

^**^ *p* < 0.01;^***^ *p* < 0.001 - comparing to control group

^##^ *p* < 0.01; ^###^ *p* < 0.001– comparing to PCP group

**Fig. 3:**
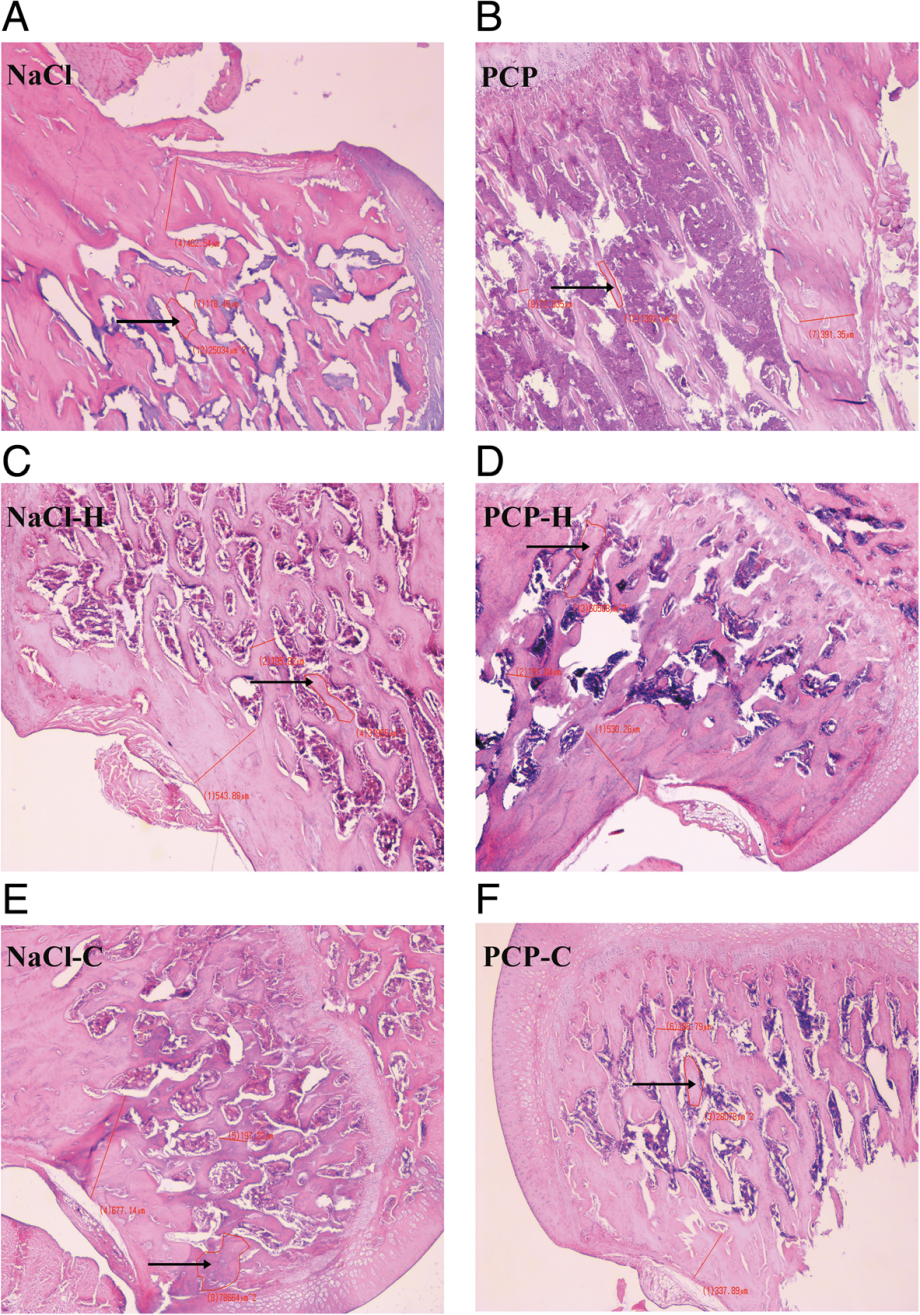
Comparative review of proximal femoral metaphysis in male rats (haematoxylin and eosin, original magnification ×40; arrows point to trabecular area). Histology of the femur in: **a**) control animals (NaCl); **b**) animals perinatally treated with phencyclidine (PCP) showing a significant reduction of trabecular area; **c**) animals perinatally treated with NaCl followed by treatment with haloperidol (NaCl-H) showing a reduction of trabecular area; **d**) animals perinatally treated with PCP followed by treatment with haloperidol (PCP-H) showing a more profound reduction of trabecular area compared to both NaCl and PCP animals; **e**) animals perinatally treated with NaCl followed by treatment with clozapine (NaCl-C) showing a significant increase of trabecular area compared to control animals; **f**) animals perinatally treated with PCP followed by treatment with clozapine (PCP-C) showing a significant increase of trabecular area compared to both NaCl and PCP animals

**Fig. 4:**
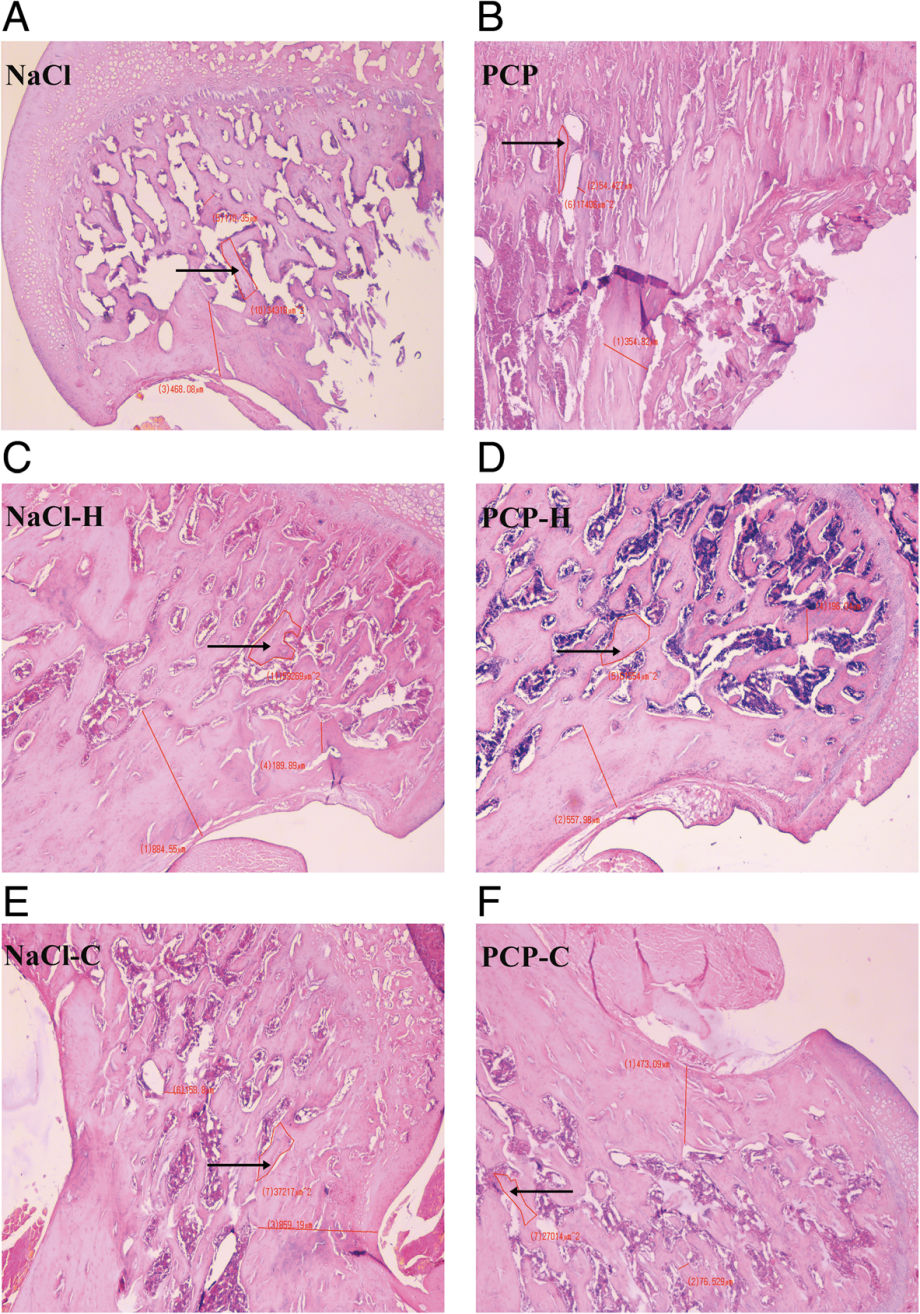
Comparative review of proximal femoral metaphysis in female rats (haematoxylin and eosin; original magnification ×40; arrows point to trabecular area). Histology of the femur in: **a**) control animals (NaCl); **b**) animals perinatally treated with phencyclidine (PCP) showing a significant reduction of trabecular area; **c**) animals perinatally treated with NaCl followed by treatment with haloperidol (NaCl-H); **d**) animals perinatally treated with PCP followed by treatment with haloperidol (PCP-H) showing a significant decrease of trabecular area compared to NaCl animals and a significant increase of trabecular area compared to PCP animals; **e**) animals perinatally treated with NaCl followed by treatment with clozapine (NaCl-C); **f**) animals perinatally treated with PCP followed by treatment with clozapine (PCP-C) showing a significant increase of trabecular area compared to both NaCl and PCP animals

Representative pictures showing optic microscopy of cortical and trabecular structure of proximal femur in male and female rats are presented on Figs. 3 and 4.

### Body weight, retroperitoneal and periepididymal fat content

Effects of perinatal PCP treatment, haloperidol and clozapine on body weight, retroperitoneal and periepididymal fat content are presented in Table 2. ANOVA detected significant body weight changes in both male [F(5,41) = 14.75, *p* < 0.001] and female [F(5,47) = 4.9, *p* < 0.001] rats. Post hoc analysis with Fisher’s test in male rats has shown a significant decrease of body weight in PCP-H (*p* < 0.001) compared to control and PCP group and a significant increase in NaCl-C group (*p* < 0.01) compared to control. In female rat’s body weight was significantly lower in PCP (*p* < 0.05) and PCP-H group (*p* < 0.01) compared to control.

**Table 2:** Body weight, retroperitoneal and periepididymal adipose tissues weights

		NaCl	PCP	NaCl-H	PCP-H	NaCl-C	PCP-C
BW (g)	M	354.2 ± 6.1	361.3 ± 6.7	338.5 ± 7.9	305 ± 7^***###^	386 ± 5^**^	342.4 ± 6.5
	F	256.7 ± 2.3	229 ± 8^*^	262 ± 6	222.4 ± 8^**^	260 ± 12	241.5 ± 5.6
RPF (g)	M	2.5 ± 0.1	3.3 ± 0.1	2.9 ± 0.3	2.8 ± 0.1	3.7 ± 0.4^**^	3.6 ± 0.4^**^
	F	2.79 ± 0.18	2.63 ± 0.37	2.51 ± 0.24	2.34 ± 0.26	3.43 ± 0.28	3.03 ± 0.14
PEF (g)	M	2.5 ± 0.3	2.7 ± 0.2	2.6 ± 0.5	2.3 ± 0.2	2.8 ± 0.4	2.7 ± 0.4

Effects of perinatal phencyclidine (PCP) treatment, haloperidol (H) and clozapine (C) on body weight (BW), retroperitoneal (RPF) and periepididymal (PEF) fat content in male (M) and female (F) rats. Results are presented as mean values with standard error of the mean (SEM)

^**^ *p* < 0.01;^***^ *p* < 0.001 - comparing to control group

^###^ *p* < 0.001– comparing to PCP group

ANOVA showed a significant changes of retroperitoneal fat [F(5,41) = 2.92, *p* < 0.05] in male rats. Post hoc analysis with Fisher’s test has shown significantly increased retroperitoneal fat in NaCl-C and PCP-C groups compared to control (*p* < 0.01) in male rats. There were no differences in retroperitoneal fat content between investigated groups [F(5,47) = 2.54, *p* > 0.05] in female rats. Also, no differences in periepididymal fat content between investigated groups [F(5,41) = 1.24, *p* > 0.05] were found.

### Changes of corticosterone, IL-6 and TNF-α concentration in the serum were sex and drug specific

**Fig. 5:**
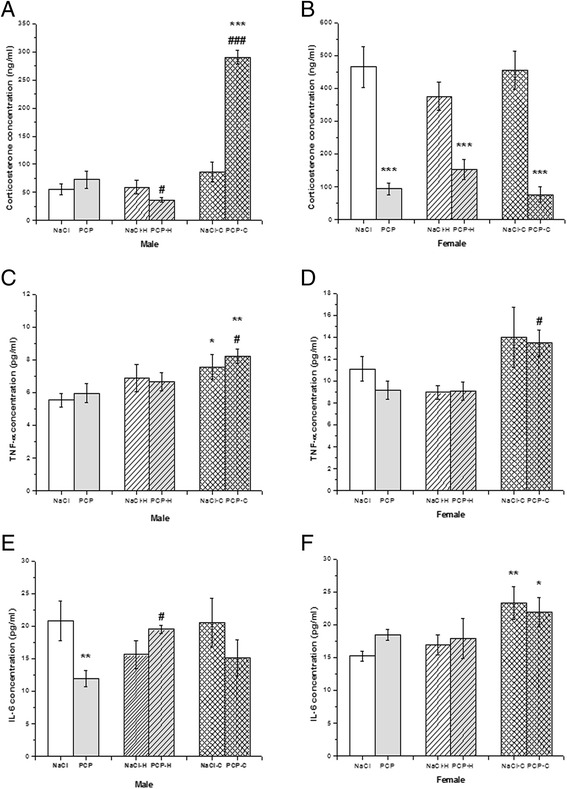
Effects of perinatal phencyclidine (PCP) treatment, haloperidol (H) and clozapine (C) on serum corticosterone, TNF-α and IL-6 concentration in male (**a**, **c** and **e**) and female (**b**, **d** and **f**) rats. Results are presented as mean values with standard error of the mean (SEM). *p < 0.05; **p < 0.01;***p < 0.001 - comparing to control group. #*p* < 0.05; ### *p* < 0.001 – comparing to PCP group

Effects of perinatal PCP treatment, haloperidol and clozapine on corticosterone, TNF-α and IL-6 concentration are presented in Fig. 5.

In male rats ANOVA showed a significant changes of corticosterone concentration [F(5,34) = 53.79, *p* < 0.001] between investigated groups. Post hoc analysis with Fisher’s test showed significantly decreased corticosterone concentration in PCP-H group (*p* < 0.05) compared to PCP group and a significantly increased corticosterone concentration in PCP-C group (*p* < 0.001) compared to both control and PCP group. In female rats [F(5,32) = 19.6, *p* < 0.001] corticosterone concentration was significantly decreased in PCP, PCP-H and PCP-C group compared to control (*p* < 0.001) (Fig. 5a and b).

ANOVA showed a significant changes of TNF-α concentration [F(5,34) = 2.84, *p* < 0.05] between investigated groups in male rats. Post hoc analysis with Fisher’s test showed significantly increased TNF-α concentration in NaCl-C group (*p* < 0.05) compared to control. Also, TNF-α concentration was significantly increased in PCP-C group compared to both control (*p* < 0.01) and PCP group (*p* < 0.05). In female rats [F(5,34) = 2.67, *p* < 0.05] the only observed change was a significant increase of TNF-α concentration in PCP-C (*p* < 0.05) group compared to PCP group (Fig. 5c and d).

ANOVA showed a significant changes of IL-6 concentration [F(5,33) = 2.11, *p* < 0.05] between investigated groups in male rats. Post hoc analysis with Fisher’s test showed significantly decreased IL-6 concentration in PCP group (*p* < 0.01) compared to control and a significantly increased IL-6 concentration in PCP-H group (*p* < 0.05) compared to PCP group. In female rats [F(5,33) = 2.55, *p* < 0.05] there was a significant increase of IL-6 concentration in NaCl-C (*p* < 0.01) and PCP-C (*p* < 0.05) group compared to control (Fig. 5e and f).

### Changes of glucose, cholesterol and triacylglycerol concentration in serum are more pronounced in male rats

Concentrations of glucose, cholesterol, triacylglycerol, calcium and phosphate are presented in Table 3. Glucose serum concentration in all experimental groups was in the reference range that is for male 5.0-12.2 mmol/L and for female 5.6-10.2 mmol/L rats. Statistically significant difference of glucose concentration between experimental groups [F(5,30) = 2.25, *p* < 0.05] was seen only in male rats where post hoc analysis has revealed significant reduction in PCP comparing to control group (*p* < 0.05).

**Table 3:** Spectrophotometric measurement of glucose, cholesterol, triacylglycerol, calcium and phosphate concentrations

		NaCl	PCP	NaCl-H	PCP-H	NaCl-C	PCP-C
Glucose (mmol/L)	M	8.34 ± 0.39	7.33 ± 0.19^*^	7.73 ± 0.27	7.60 ± 0.32	8.61 ± 0.49	8.08 ± 0.13
	F	7.46 ± 0.29	7.17 ± 0.30	7.68 ± 0.21	7.63 ± 0.33	7.74 ± 0.22	7.85 ± 0.33
Cholesterol (mmol/L)	M	1.00 ± 0.02	1.18 ± 0.06^*^	1.25 ± 0.05^***^	1.28 ± 0.05^***^	1.36 ± 0.04^***^	1.26 ± 0.06^***^
	F	1.93 ± 0.17	1.65 ± 0.05^*^	1.72 ± 0.07	1.53 ± 0.06^**^	1.84 ± 0.07	1.68 ± 0.08^*^
Triacylglycerol (mmol/L)	M	1.98 ± 0.14	1.73 ± 0.09	1.73 ± 0.09	1.50 ± 0.15^*^	1.37 ± 0.14^**^	1.43 ± 0.18^**^
	F	1.24 ± 0.08	1.37 ± 0.12	1.39 ± 0.12	1.16 ± 0.11	1.16 ± 0.11	1.25 ± 0.11
Calcium (mmol/L)	M	2.38 ± 0.05	2.4 ± 0.06	2.28 ± 0.06	2.32 ± 0.05	2.3 ± 0.07	2.33 ± 0.05
	F	2.25 ± 0.06	2.27 ± 0.07	2.33 ± 0.07	2.33 ± 0.05	2.27 ± 0.05	2.28 ± 0.06
Phosphate (mmol/L)	M	3.17 ± 0.09	3.42 ± 0.18	3.15 ± 0.04	3.32 ± 0.09	3.27 ± 0.17	3 ± 0.19
	F	2.82 ± 0.17	3.05 ± 0.16	3.23 ± 0.14	2.9 ± 0.11	2.7 ± 0.18	2.73 ± 0.08

Effects of perinatal phencyclidine (PCP) treatment, haloperidol (H) and clozapine (C) on glucose, cholesterol, triacylglycerol, calcium and phosphate serum concentrations in male (M) and female (F) rats. Results are presented as mean values with standard error of the mean (SEM)

^*^ *p* < 0.05; ^**^ *p* < 0.01;^***^ *p* < 0.001 - comparing to control group

The concentration of cholesterol in the tested groups was in the normal ranges both in males (1.8-3.6 mmol/L) and females (1.1-4.1 mmol/L). However, the significant differences between groups were seen in both males [F(5,30) = 6.48, *p* < 0.001] and females [F(5,30) = 2.41, *p* < 0.05]. It is interesting that in all experimental groups of males cholesterol concentrations were significantly increased (*p* < 0.05 for PCP group, *p* < 0.001 for NaCl-H, PCP-H, NaCl-C and PCP-C groups) compared to control, while in female rats the concentrations were significantly decreased in PCP (*p* < 0.05), PCP-H (*p* < 0.01) and PCP-C (*p* < 0.05) groups compared to control.

The concentrations of triacylglycerol in all experimental groups were also in the reference range for males (0.7-5.12 mmol/L) and females (0.4-5.92 mmol/L). Significant difference in the concentration of triglycerides between tested groups was seen only in male rats [F(5,30) = 2.78, *p* < 0.05] where by applying post hoc analysis, a statistically significant reduction was demonstrated in the PCP-H group (*p* < 0.05) and a highly statistically significant reduction in the NaCl-C and PCP-C groups (*p* < 0.01) compared to the control group.

ANOVA did not show significant changes of calcium concentration in male rats [F(5,30) = 0.67, *p* > 0.05] nor in female rats [F(5,30) = 0.37, *p* > 0.05] between investigated groups. Also, concentration of phosphate was not changed in male rats [F(5,30) = 1.09, *p* > 0.05] and female rats [F(5,30) = 1.97, *p* > 0.05] between investigated groups.

## Discussion

In this study, the influence of haloperidol and clozapine treatment on the bone, body composition, corticosterone, proinflammatory cytokines and metabolic parameters was investigated in male and female rats perinatally treated with PCP. The DXA measurements were done at PN 60 and PN 98 and the dynamic of changes caused by the treatment with antipsychotics was followed. Changes in BMD and BMC in treated groups were more pronounced on PN 98.

Reduction of BMD and especially BMC, as a more precise indicator for osteoporosis and risk factor for bone fractures [51], clearly indicates that perinatal use of PCP in rats, representing an animal model of schizophrenia, has long-term effects on bones. This is in accordance with findings in patients with schizophrenia. Recent meta-analyses have suggested that BMD is significantly lower in schizophrenia patients than in healthy controls [52] and that people with schizophrenia are at increased risk of developing fractures [53]. The finding of decreased bone density in our PCP perinatally treated rats indicate that complex changes in the brain, that are the basis for schizophrenia pathophysiology, could be responsible for observed changes of bones. It is known that bone remodelling which is essential for bone acquisition and bone maintenance is regulated systemically by many hormones and locally by cytokines, but recently the regulation through a central – beta adrenergic system relay [54] has been suggested. Also, the role of brain serotonin, in the modulation of sympathetic nervous system outflow to the skeleton and regulation of bone remodelling in experimental animals has been suggested [55, 56]. Our previous results showing normal prolactin levels in PCP perinatally treated rats [57], as well as, the changes of corticosterone or IL-6 and TNF-α concentration in this study, indicate that these factors are not responsible for the changes observed on bones. Also, findings of normal serum concentration of calcium and phosphates in our animals indirectly indicate normal levels of hormones involved in calcium homeostasis.

The investigations of the influence of antipsychotics in our model have revealed sex differences in the effects of haloperidol and the absence of the effects of clozapine on bones regardless the sex, in control (NaCl perinatally treated) animals. Among these animals decrease of total BMD and BMC was only seen in male haloperidol treated rats. It seems that observed changes are not the consequence of the alterations of corticosterone, IL-6 and TNF-α concentration since no change of these factors was observed. Previously, we have demonstrated dose dependent effects of haloperidol treatment on prolactin levels in male rats perinatally treated either with NaCl or PCP [57] and that higher dose of haloperidol (3 mg/kg) markedly increased prolactin levels while dose of haloperidol (1 mg/kg) used in the present investigation did not cause changes in level of prolactin. Therefore, this hormone cannot be regarded as a causal factor for the bone changes observed in the present study. The effects of haloperidol on BMD and BMC were present in PCP perinatally treated animals both male and female rats. The haloperidol treatment was related to the further deterioration of bone density that was already significantly decreased in PCP group without any antipsychotic medication. However, this further decrease was more significant in male rats. Treatment with clozapine, on the other hand, did not cause further deterioration of BMD and BMC in comparison to PCP perinatally treated rats and even modest protective effect of this drug was seen by microscopic examination in animals of both sexes. Interesting are the findings of corticosterone and interleukin measurements in these animals. While haloperidol did not cause changes of corticosterone, IL-6 and TNF-α concentrations both in NaCl and PCP perinatally treated rats comparing to control groups, clozapine treatment caused the significant elevation of IL-6 in female rats and significant elevation of TNF-α in male rats in both NaCl and PCP perinatally treated animals (NaCl-C and PCP-C groups) compared to controls. Also, clozapine treatment caused significant elevation of corticosterone concentration in male and significant decrease in female PCP perinatally treated rats. Our previous investigation has revealed the absence of the effects of clozapine on prolactin concentration regardless of the treatment dose.

Gender differences of antipsychotic effects on bone [17] were frequently analyzed in patients with schizophrenia. Some studies have demonstrated the beneficial effects of PS antipsychotics [58] and the association of PR antipsychotics with hip fractures [59] in both genders. Lin et al. [60] have found dose-related bone-density protecting effect of clozapine in women with chronic schizophrenia. However, although the prevalence of hyperprolactinemia was more frequently seen in women taking PR antipsychotics [61–63] the meta-analysis done by Stubbs et al. [16], that has included nineteen studies and more than three thousands patients with schizophrenia, pointed out that men are more vulnerable to osteoporosis and osteopenia than women. This finding is in accordance with our results. Interestingly, Lee et al. [64] have suggested that decreased bone mineral density found in the male schizophrenia patients may be caused rather by the negative schizophrenia symptoms than the hyperprolactinemia due to the antipsychotics.

Sex specific effects of PCP on cognition, brain derived neurotrophic factor (BDNF), spine synapses and dopamine turnover in prefrontal cortex were found in the juvenile but not, adult monkey’s representing the primate PCP model of schizophrenia. Namely in male juvenile monkey’s grater loss of spine synapses, grater effects on cognition, lower level of dopamine turnover and lower level of BDNF were found after two weeks of treatment with intramuscular injections of PCP [65]. As the most straightforward explanation for observed sex-dependent effects of PCP the authors have suggested the findings of protective and reparative effects of estradiol and progesterone in the CNS [66, 67] including the finding of the presence of an estrogen response element-like motif in the BDNF gene that regulates BDNF expression levels [68]. In accordance with these results is also our finding that at the PN 60 (juvenile rats) the effects of PCP on BMD and BMC are present only in male rats.

Recently two estrogen hypotheses in SCH have been proposed [69]. First one is the hypoestrogenism or deficiency hypothesis which describes gonadal dysfunction in women with SCH. The second is the protection hypothesis which states that estrogen exerts a relative protection against SCH in premenopausal women. Our results of selective sensitivity of PCP perinatally treated females to haloperidol treatment could be related to hypoestrogenism. It is possible that control (NaCl perinatally pre-treated) females in our study are protected against effects of haloperidol on bones, seen in healthy male rats, by their normal level of estrogen and that PCP itself produces hypoestrogenism that is further pronounced by haloperidol treatment. Encouraging for this assumption is the finding that estrogen pre-treatment is effective in attenuation of a PCP-induced object recognition memory deficit in rats [70].

Effects of antipsychotics on experimental animals were investigated in several studies [38, 39]. To the best of our knowledge only one study assessed effect of risperidone in an animal model of schizophrenia showing protective effects of long-term treatment with atypical antipsychotic [44].

Study of Kunimatsu et al. [39] has indicated that chronic treatments with typical antipsychotics haloperidol or chlorpromazine induced a loss of trabecular bone of the femur in female rats. These results differ to results demonstrated in this study and could represent consequence of the applied doses as authors have used 2 and 10 mg/kg/day of haloperidol that highly exceed the doses applied in human treatment.

On the other hand, Costa et al. [38] have demonstrated, by both DXA and micro-computed tomography, that 6 weeks of clozapine, but not haloperidol treatment, decreases BMD in growing male rats. The authors have suggested that this effect may be mediated by direct actions of clozapine to decrease proliferation and differentiation of osteoblasts. Interestingly, our results show that clozapine treatment leads to normalization of the changes caused by perinatal use of PCP and, as we have previously mentioned, haloperidol is more harmful. The possible reason for the discrepancy between findings of these studies could be in the use of different protocol, especially the dose and duration of the antipsychotic treatment. In the study done by Costa et al. [38], the drugs were administered daily in the form of s.c. injection for 42 days, in the lower doses comparing to our protocol and this might have impact to antipsychotic metabolism and effects of the drugs. Also, the authors did not analyse the influence of antipsychotics in an animal model of schizophrenia.

Contrary to the effects on parameters measured on bones, the effects on biochemical parameters in serum were more pronounced after the treatment with clozapine. The corticosterone levels were higher in female than in male control (NaCl perinatally treated) rats and these finding is in accordance with the results of Ableson et al. [71] who have investigated plasma corticosterone levels during automated blood sampling in rats. Perinatal PCP treatment in our study was followed by the decrease of serum corticosterone concentration in female rats and decrease of IL-6 concentration in male rats. The study of Amani et al. [72] has indicated that neonatal PCP treatment did not alter baseline corticosterone levels both in male and female mice, but these authors have obtained experimental evidence showing that stress dose of PCP (10 mg/kg) increases corticosterone levels, anxiety- and depression-related behaviours in males, while decreases levels of anxiety without any significant effect on depression in female mice in adulthood.

The treatment with haloperidol in present study was followed by the decrease of corticosterone levels both in males and females perinatally treated with PCP, but clozapine has shown different effects in males and females. Highly significant increase of corticosterone level was seen in male rats while in female rats this drug did not have further influence on the changes already produced by PCP.

Elevated cortisol concentrations are found in first-episode, drug-naive schizophrenia patients without influence of antipsychotic medication, as well as in chronic medicated patients [19]. Also, patients with schizophrenia frequently display an impaired HPA axis response following an acute stress [73–77]. Both first and second generation antipsychotics have been associated with cortisol changes in patients with psychosis, but increasing evidences suggest that second generation antipsychotics reduce cortisol to a greater extent comparing to first generation [78–80]. The study, conducted in a small sample of healthy volunteers [81], has demonstrated no effect of the haloperidol on cortisol levels, and a reduction of cortisol levels by antipsychotics like quetiapine and risperidone. The effect of second generation antipsychotics on cortisol has been suggested to reflect differences in affinity and occupancy at the D2 and 5-HT receptor subtypes [82].

Increasing evidences indicate immune pathway dysfunction in schizophrenia. Autoimmune disorders and infections are associated with increased risk of developing schizophrenia [83, 84]. However, measurements of the levels of pro-inflammatory cytokines have obtained different results. Elevated levels are found in high risk and first episode psychosis patients [85, 86] and also during symptom exacerbations and stable phases of chronic illness [87, 88]. Dunjić et al. [89] have demonstrated higher IL-6 and lower TNF-α level in patients with schizophrenia in both exacerbation and remission phase in comparison to healthy controls and the absence of significant correlation between the levels of cytokines and sex, age, BMI, smoking habits, antipsychotic medication, duration of treatment and duration of illness. Chase et al. [90] have found that the levels of IL-6 mRNA in the peripheral blood mononuclear cells, in the absence of any other information, reliably discriminated between a diagnosis of schizophrenia and normal controls suggesting it as a useful and easily clinically accessible biomarker for the diagnosis of schizophrenia. Genome-wide association studies have identified immune system gene loci as among the leading associations with schizophrenia [91–94]. Chiappelli et al., [95] have found dysfunction in the homeostatic interactions between glucocorticoid and immune pathways. The applied stress paradigm has induced a rise in both cortisol and IL-6 in SCH patients, while in healthy controls a more robust acute cortisol response followed by a steeper decline of IL-6 that corresponds to the expected anti-inflammatory effects of cortisol, was seen. The opposite relationship demonstrated in SCH patients suggested a presence of an inability to down-regulate inflammatory responses to psychological stress. Our results have shown that perinatal PCP treatment does not cause significant changes in the measured pro-inflammatory cytokine concentrations. The only difference that has been noticed was the decrease of IL-6 in male rats. Treatment with haloperidol was not followed by significant changes of IL-6 and TNF-α both in male and female rats regardless of perinatal treatment, while clozapine caused significant elevation of TNF-α in male and significant elevation of both IL-6 and TNF-α in females regardless of perinatal treatment. In accordance with our findings is the study of Handley et al. [96] demonstrating that haloperidol, but not aripiprazole, lowers cortisol and IL-6 levels, and increases hippocampal regional cerebral blood flow within hours of drug administration. In the study of O’Connell et al. [97] increased levels of proinflammatory cytokines and BMI were found in female, but not male patients treated with clozapine compared to healthy controls. The authors have suggested that association of increased number of adipocytes may contribute to increased cytokine serum concentrations in females.

Fonseka et al. [98] have proposed that antipsychotics with a high propensity for weight gain, such as clozapine and olanzapine, influence the expression of immune genes, and induce changes in serum cytokine levels to ultimately down-regulate neuroinflammation. Inflammatory cytokines are normally involved in anorexigenic responses and reduced inflammation has been shown to mediate changes in feeding behaviours and other metabolic parameters, resulting in obesity. Study on rats [99] however, has shown that clozapine induces an immune response with an increase in inflammatory cytokines such as IL-6 due to its oxidation to a reactive nitrenium ion that covalently binds to neutrophils leading to the increase of inflammatory cytokines.

Increase of body weight and retroperitoneal fat in male rats treated with clozapine, seen in our study, could be related to increased serum corticosterone. Hyperactivity of the HPA axis is generally associated with obesity [100–102]. However, the increase of corticosterone was seen only in male PCP-C group while changes of body weight and retroperitoneal fat were observed in both NaCl-C and PCP-C groups of males indicated that increase of corticosterone could not be the only factor responsible clozapine influence on these parameters. The sex specific effects of clozapine were also demonstrated by Baptista et al. [103] who have found that different doses of clozapine (from 0.5 – 20 mg) applied intraperitoneally for 21 days have effects on body weight only in male rats. On the contrary Ferno et al. [104] show that depot injections of 100–250 mg/kg olanzapine in male rats resulted in weight loss rather than weight gain. Investigation of the ability of clozapine to induce weight gain in female rats [41], with progressively lowered doses of the drug, have resulted in finding that clozapine did not induce weight gain despite the given doses. Instead, clozapine induced weight loss without alterations in food intake and muscle mass or changes in glucose, insulin, leptin and prolactin levels. In clinical studies, substantial clozapine induced weight gain was observed in humans [105]. In the meta-analysis performed by Gressier et al. [106] a strong relationship between serotonergic genes polymorphism and clinical response to clozapine was seen without clear findings of influence of tested genes polymorphism on clozapine induced weight gain.

In this study, significant increase of cholesterol concentration in male rats compared to control animals was shown in all experimental groups. It is noteworthy to mention that although those significant differences were noticed, all values have remained within the reference range for this kind of laboratory animals. Sex-dependent metabolic alterations have been also manifested in rat liver after 12-week exposition to haloperidol or clozapine [107]. The stress of endoplasmic reticulum caused by applied drugs via changes in Ca^2+^ homeostasis, is thought to be responsible for observed changes. It seems that women were protected by their estrogen. A variety of preclinical and clinical studies have indicated that in animals and patients treated with clozapine and haloperidol, obesity and elevated cholesterol and triacylglycerol’s concentrations are due to the changes in the expression of genes involved in lipogenesis [108, 109], as well as in disorders of hormones responsible for the metabolism of fat [107, 110]. Olanzapine applied as intramuscular injection in the dose of 200 mg/kg or above has induced significant elevation of plasma cholesterol levels and pronounce activation of lipogenic gene expression in the liver [104].

Limitations of the study: Antipsychotics were used in one dose and applied orally giving the possibility for various ingestions of drugs by animals. Although this can mimic the situation with schizophrenia patients that have various medication compliance, different doses and different route of application of antipsychotics are needed. Also, since the female rats were investigated, the limitation of this study lies in the fact that the phase of menstrual cycle is not monitored. Furthermore, advanced investigations should be directed to the molecular mechanism of antipsychotic influence including measuring of parathyroid hormone, vitamin D, calcitonin, RANK (receptor activator of nuclear factor kappa-B)/RANK ligand and osteoprotegerin molecules. Mechanistic approach should also include the haloperidol-treated group receiving an antiresorptive (e.g., bisphosphonates) or osteoanabolic agent (e.g., calcitonin).

## Conclusions

To the best of our knowledge, this is the first study that investigated the effects of haloperidol and clozapine on bone mass and body composition in PCP animal model of schizophrenia. Our results have shown that perinatal PCP administration causes a significant decrease in bone mass and deterioration in bone quality in male and female rats indicating that disease per se could be related to the changes of bones. Haloperidol had deleterious, while clozapine had protective effect on bones. The effects of haloperidol on bones were more pronounced in male rats. It seems that observed changes are not the consequence of the alterations of corticosterone, IL-6 and TNF-α concentration since no change of these factors was observed. Treatment with clozapine was followed by more prominent, mainly sex specific, changes of measured biochemical and metabolic parameters. Clozapine induced increase of body weight and retroperitoneal fat in male rats regardless of perinatal treatment. However, the increase of corticosterone, seen only in male PCP perinatally treated rats that received clozapine, indicate that corticosterone is not the main factor responsible for clozapine influence on body weight. Furthermore, clozapine treatment caused increase in pro-inflammatory cytokines with sex specific changes that can fit in the theory of ‘cytokine signature’. Male rats have reacted to all applied drugs by significant increase of cholesterol concentration.

Taken together our findings confirm that antipsychotics have complex influence on bone and metabolism. The use of animal models in this investigation is important since they offer a precise control of factors that are highly variable and hardly controlled in humans. Evaluation of potential markers for individual risk of antipsychotics induced adverse effects could be valuable for improvement of therapy of this life-long lasting disease.

## Additional files

Additional file 1: Table S1. Fat content measured by DXA. (DOCX 20 kb)

Additional file 2: Table S2. Lean content measured by DXA. (DOCX 19 kb)

## Abbreviations

ANOVA: The Analysis Of Variance
BMC: Bone mineral content
BMD: Bone mineral density
C: Clozapine
DXA: Dual X-ray absorptiometry
H: Haloperidol
HPA: Hypothalamic–pituitary–adrenal
Il-6: Interleukin 6
NMDA: N-methyl-D-aspartate
NRG-1: Neuregulin-1
PCP: Phencyclidine
PN: Postnatal day
RANK: Receptor activator of nuclear factor kappa-B
s.c.: Subcutaneously
S.E.M.: Standard error of the mean
SCH: Schizophrenia
TNF-α: Tumor necrosis factor alpha

## References

[1] Van Os J, Kapur S (2009). Schizophrenia. Lancet.

[2] American Psychiatric Association. Diagnostic and Statistical Manual of Mental Disorders. Fifth Edition. Arlington: American Psychiatric Association Publishing; 2013.

[3] Mueser KT, McGurk SR (2004). Schizophrenia. Lancet.

[4] Freedman R (2003). Schizophrenia. N Engl J Med.

[5] Reid IR (2008). Relationships between fat and bone. Osteoporos Int.

[6] Ballon J, Pajvani U, Freyberg Z, Leibel R, Lieberman J (2014). Molecular pathophysiology of metabolic effects of antipsychotic medications. Trends Endocrinol Metab.

[7] Pouwels S, van Staa TP, Egberts AC, Leufkens HG, Cooper C, de Vries F (2009). Antipsychotic use and the risk of hip/femur fracture: a population-based case-control study. Osteoporos Int.

[8] Maric N, Popovic V, Jasovic-Gasic M, Pilipovic N, van Os J (2005). Cumulative exposure to estrogen and psychosis: a peak bone mass, case–control study in first-episode psychosis. Schizophr Res.

[9] Mitchell AJ, Vancampfort D, De Herdt A, Yu W, De Herdt MI (2013). The prevalence of metabolic syndrome and metabolic abnormalities increased in early schizophrenia? A comparative meta-analysis of first episode, untreated and treated patients. Schizophr Bull.

[10] Naidoo U, Goff DC, Klibanski A (2003). Hyperprolactinemia and bone mineral density: the potential impact of antipsychotic agents. Psychoneuroendocrinology.

[11] Altındağ Ö, Altındağ A, Vırıt O, Savaş HA, Yılmaz M, Bozgeyik Ö (2009). Antipsikotik ilaç kullanan şizofreni hastalarında kemik mineral yoğunluğu. Klinik Psikofarmakoloji Bülteni.

[12] Jung DU, Conley RR, Kelly DL, Kim DW, Yoon SH, Jang JH (2006). Prevalence of bone mineral density loss in Korean patients with schizophrenia: a cross-sectional study. J Clin Psychiatry.

[13] Meaney AM, Smith S, Howes OD, O’Brien M, Murray RM, O’Keane V (2004). Effects of long-term prolactin-raising antipsychotic medication on bone mineral density in patients with schizophrenia. Br J Psychiatry.

[14] Liu-Seifert H, Kinon BJ, Ahl J, Lamberson S (2004). Osteopenia associated with increased prolactin and aging in psychiatric patients treated with prolactin-elevating antipsychotics. Ann N Y Acad Sci.

[15] Bulut SD, Bulut S, Tüzer V, Ak M, Ak E, Kisa C (2014). The effects of Prolactin-raising and Prolactin-sparing antipsychotics on Prolactin levels and bone mineral density in schizophrenic patients. Archives of. Neuropsychiatry.

[16] Stubbs B, De Hert M, Sepehry AA, Correll CU, Mitchell AJ, Soundy A (2014). A meta-analysis of prevalence estimates and moderators of low bone mass in people with schizophrenia. Acta Psychiatr Scand.

[17] Chen CY, Lane HY, Lin CH (2016). Effects of antipsychotics on bone mineral density in patients with schizophrenia: gender differences. Clin Psychopharmacol Neurosci.

[18] Halbreich U, Palter S (1996). Accelerated osteoporosis in psychiatric patients: possible pathophysiological processes. Schizophr Bull.

[19] Bradley AJ, Dinan TG (2010). A systematic review of hypothalamic-pituitary adrenal axis function in schizophrenia: implications for mortality. J Psychopharmacol.

[20] Aiello G, Horowitz M, Hepgul N, Pariante CM, Mondelli V (2012). Stress abnormalities in individuals at risk for psychosis: a review of studies in subjects with familial risk or with “at risk” mental state. Psychoneuroendocrinology.

[21] Borges S, Gayer-Anderson C, Mondelli V (2013). A systematic review of the activity of the hypothalamic-pituitary-adrenal axis in first episode psychosis. Psychoneuroendocrinology.

[22] Miller BJ, Buckley P, Seabolt W, Mellor A, Kirkpatrick B (2011). Meta-analysis of cytokine alterations in schizophrenia: clinical status and antipsychotic effects. Biol Psychiatry.

[23] Mondelli V, Dazzan P, Hepgul N, Di Forti M, Aas M, D'Albenzio A (2010). Abnormal cortisol levels during the day and cortisol awakening response in first-episode psychosis: the role of stress and of antipsychotic treatment. Schizophr Res.

[24] Jones CA, Watson DJ, Fone KC (2011). Animal models of schizophrenia. Br J Pharmacol.

[25] Radonjic NV, Knezevic ID, Vilimanovich U, Kravic-Stevovic T, Marina LV, Nikolic T (2010). Decreased glutathione levels and altered antioxidant defence in an animal model of schizophrenia: long-term effects of perinatal phencyclidine administration. Neuropharmacology.

[26] Wang C, McInnis J, Ross-Sanchez M, Shinnick-Gallagher P, Wiley JL, Johnson KM (2001). Long-term behavioural and neurodegenerative effects of perinatal phencyclidine administration: implications for schizophrenia. Neuroscience.

[27] Bey T, Patel A (2007). Phencyclidine intoxication and adverse effects: a clinical and pharmacological review of an illicit drug. Cal J Emerg Med.

[28] Olney JW, Farber NB (1995). Glutamate receptor dysfunction and schizophrenia. Arch Gen Psychiatry.

[29] McKibben CE, Reynolds GP, Jenkins TA (2016). Concurrent Risperidone administration attenuates the development of Locomotor sensitization following sub-chronic phencyclidine in rats. Pharmacopsychiatry.

[30] Andersen JD, Pouzet B (2004). Spatial memory deficits induced by perinatal treatment of rats with PCP and reversal effect of D-serine. Neuropsychopharmacology.

[31] Wang C, McInnis J, West JB, Bao J, Anastasio N, Guidry JA (2003). Blockade of phencyclidine induced cortical apoptosis and deficits in pre pulse inhibition by M40403, a superoxide dismutase mimetic. J Pharmacol Exp Ther.

[32] Mouri A, Noda Y, Enomoto T, Nabeshima T (2007). Phencyclidine animal models of schizophrenia: approaches from abnormality of glutamatergic neurotransmission and neurodevelopment. Neurochem Int.

[33] Grayson B, Barnes SA, Markou A, Piercy C, Podda G, Neill JC (2016). Postnatal phencyclidine (PCP) as a Neurodevelopmental animal model of schizophrenia Pathophysiology and Symptomatology: a review. Curr Top Behav Neurosci.

[34] Radonjić NV, Petronijević ND, Vučković SM, Prostran MS, Nešic ZI, Todorović VR (2008). Baseline temperature in an animal model of schizophrenia: long-term effects of perinatal phencyclidine administration. Physiol Behav.

[35] Stojković T, Radonjić NV, Velimirović M, Jevtić G, Popović V, Doknić M (2012). Risperidone reverses phencyclidine induced decrease in glutathione levels and alterations of antioxidant defence in rat brain. Progr Neuropsychopharmacol Biol Psychiatr.

[36] Radonjić NV, Jakovcevski I, Bumbaširević V, Petronijević ND (2013). Perinatal phencyclidine administration decreases the density of cortical interneurons and increases the expression of neuregulin-1. Psychopharmacology.

[37] Jevtić G, Nikolić T, Mirčić A, Stojković T, Velimirović M, Trajković V (2016). Mitochondrial impairment, apoptosis and autophagy in a rat brain as immediate and long-term effects of perinatal phencyclidine treatment - influence of restraint stress. Prog Neuro-Psychopharmacol Biol Psychiatry.

[38] Costa JL, Smith G, Watson M, Lin JM, Callon K, Gamble G (2011). The atypical anti psychotic clozapine decreases bone mass in rats in vivo. Schizophr Res.

[39] Kunimatsu T, Kimura J, Funabashi H, Inoue T, Seki T (2010). The antipsychotics haloperidol and chlorpromazine increase bone metabolism. Regul Toxicol Pharmacol.

[40] Motyl KJ, Dick-de-Paula I, Maloney AE, Lotinun S, Bornstein S, de Paula FJ (2012). Trabecular bone loss after administration of the second-generation antipsychotic risperidone is independent of weight gain. Bone.

[41] Cooper GD, Harrold JA, Halford JC, Goudie AJ (2008). Chronic clozapinee treatment in female rats does not induce weight gain or metabolic abnormalities but enhances adiposity: implications for animal models of antipsychotic-induced weight gain. Prog Neuro-Psychopharmacol Biol Psychiatry.

[42] Smith GC, Chaussade C, Vickers M, Jensen J, Shepherd PR (2008). Atypical antipsychotic drugs induce derangements in glucose homeostasis by acutely increasing glucagon secretion and hepatic glucose output in the rat. Diabetologia.

[43] Volpato AM, Zugno AL, Quevedo J (2013). Recent evidence and potential mechanisms underlying weight gain and insulin resistance due to atypical antipsychotics. Rev Bras Psiquiatr.

[44] Petronijevic N, Sopta J, Doknic M, Radonjic N, Petronijevic M, Pekic S (2013). Chronic risperidone exposure does not show any evidence of bone mass deterioration in animal model of schizophrenia. Prog Neuro-Psychopharmacol Biol Psychiatry.

[45] Adams SM, de RiveroVaccari JC, Corriveau RA (2004). Pronounced cell death in the absence of NMDA receptors in the developing somatosensory thalamus. J Neurosci.

[46] Ikonomidou C, Bosch F, Miksa M, Bittigau P, Vöckler J, Dikranian K (1999). Blockade of NMDA receptors and apoptotic neurodegeneration in the developing brain. Science.

[47] Kapur S, VanderSpek SC, Brownlee BA, Nobrega JN (2003). Antipsychotic dosing in preclinical models is often unrepresentative of the clinical condition: a suggested solution based on in vivo occupancy. J Pharmacol Exp Ther.

[48] Schotte A, Janssen PF, Gommeren W, Luyten WH, Van Gompel P, Lesage AS (1996). Risperidone compared with new and reference antipsychotic drugs: in vitro and in vivo receptor binding. Psychopharmacology.

[49] Steward LJ, Kennedy MD, Morris BJ, Pratt JA (2012). Chronic phencyclidine (PCP)-induced modulation of muscarinic receptor mRNAs in rat brain: impact of antipsychotic drug treatment. Neuropharmacology.

[50] Terry AV, Gearhart DA, Warner SE, Zhang G, Bartlett MG, Middlemore ML (2007). Oral haloperidol or risperidone treatment in rats: temporal effects on nerve growth factor receptors, cholinergic neurons, and memory performance. Neuroscience.

[51] Riggs BL, Wahner HW, Dunn WL, Mazess RB, Offord KP, Melton LJ (1981). Differential changes in bone mineral density of the appendicular and axial skeleton with aging: relationship to spinal osteoporosis. J Clin Invest.

[52] Tseng PT, Chen YW, Yeh PY, KY T, Cheng YS, Bone Mineral WCK (2015). Density in schizophrenia: an update of current meta-analysis and literature review under guideline of PRISMA. Medicine (Baltimore).

[53] Stubbs B, Gaughran F, Mitchell AJ, De Hert M, Farmer R, Soundy A (2015). Schizophrenia and the risk of fractures: a systematic review and comparative meta-analysis. Gen Hosp Psychiatry.

[54] Bonnet N, Pierroz DD, Ferrari SL (2008). Adrenergic control of bone remodeling and its implications for the treatment of osteoporosis. J Musculoskelet Neuronal Interact.

[55] Kawai M, Rosen CJ (2010). Minireview: a skeleton in serotonin’s closet?. Endocrinology.

[56] Quednow BB, Geyer MA, Halberstadt AL (2009). Serotonin and schizophrenia. Müller CR, Jacobs B. Handbook of the behavioral neurobiology of serotonin. London.

[57] Nikolic T, Nenadovic M, Jevtic G, Stojkovic T, Velimirovic M, Lazovic M (2016). Dose dependent effects of antipsychotics on prolactin and corticosterone concentration in a phencyclidine animal model of schizophrenia. Eur Neuropsychopharmacol.

[58] Lin CH, Lin CY, Huang TL, Wang HS, Chang YC, Lane HY (2015). Sex-specific factors for bone density in patients with schizophrenia. Int Clin Psychopharmacol.

[59] Takahashi T, Uchida H, John M, Hirano J, Watanabe K, Mimura M (2013). The impact of prolactin-raising antipsychotics on bone mineral density in patients with schizophrenia: findings from a longitudinal observational cohort. Schizophr Res.

[60] Lin CH, Huang KH, Chang YC, Huang YC, Hsu WC, Lin CY (2012). Clozapine protects bone mineral density in female patients with schizophrenia. Int J Neuropsychopharmacol.

[61] Bushe C, Shaw M, Peveler RCA (2008). Review of the association between antipsychotic use and hyperprolactinaemia. J Psychopharmacol.

[62] Kinon BJ, Liu-Seifert H, Stauffer VL, Jacob J (2013). Bone loss associated with hyperprolactinemia in patients with schizophrenia. Clin Schizophr Relat Psychoses.

[63] Kinon BJ, Gilmore JA, Liu H, Halbreich UM (2003). Hyperprolactinemia in response to antipsychotic drugs: characterization across comparative clinical trials. Psychoneuroendocrinology.

[64] Lee TY, Chung MY, Chung HK, Choi JH, Kim TY, So HS. Bone density in chronic schizophrenia with long-term antipsychotic treatment: preliminary study. Psychiatry Investig 2010;7: 278-284. 10.4306/pi.2010.7.4.278 PMC3022315 21253412

[65] Elsworth JD, Groman SM, Jentsch JD, Leranth C, Redmond DE Jr, Kim JD, Diano S, Roth RH. Primate phencyclidine model of schizophrenia: sex-specific effects on cognition, brain derived Neurotrophic factor, spine synapses, and dopamine turnover in prefrontal cortex. Int J Neuropsychopharmacol. 2014;18(6). doi: 10.1093/ijnp/pyu048. 10.1093/ijnp/pyu048 PMC4438537 25522392

[66] Azcoitia I, Arevalo MA, De Nicola AF, Garcia-Segura LM (2011). Neuroprotective actions of estradiol revisited. Trends Endocrinol Metab.

[67] Singh M, Su C (2013). Progesterone and neuroprotection. Horm Behav.

[68] Sohrabji F, Miranda RC, Toran-Allerand CD (1995). Identification of a putative estrogen response element in the gene encoding brain-derived neurotrophic factor. Proc Natl Acad Sci U S A.

[69] Riecher-Rossler A (2002). Oestrogen effects in schizophrenia and their potential therapeutic implications—review. Arch Womens Ment Health.

[70] Sutcliffe JS, Rhaman F, Marshall KM, Neill JC (2008). Oestradiol attenuates the cognitive deficit induced by acute phencyclidine treatment in mature female hooded-Lister rats. J Psychopharmacol.

[71] Abelson KS, Adem B, Royo F, Carlsson HE, Hau J (2005). High plasma Corticosterone levels persist during frequent automatic blood sampling in rats. In Vivo.

[72] Amani M, Samadi H, Doosti MH, Azarfarin M, Bakhtiari A, Majidi-Zolbanin N (2013). Neonatal NMDA receptor blockade alters anxiety- and depression-related behaviours in a sex-dependent manner in mice. Neuropharmacology.

[73] Albus M, Ackenheil M, Engel RR, Müller F (1982). Situational reactivity of autonomic functions in schizophrenic patients. Psychiatry Res.

[74] Jansen LMC, Gispen-de Wied CC, Khan RS (2000). Selective impairments in the stress response in schizophrenia patients. Psychopharmacology.

[75] Goldman MB, Gnerlich J, Hussain M (2007). Neuroendocrine responses to a cold pressor stimulus in polydipsic hyponatremic and in matched schizophrenic patients. Neuropsychopharmacology.

[76] Brenner K, Liu A, Laplante DP, Lupien S, Pruessner JC, Ciampi A (2009). Cortisol response to a psychosocial stressor in schizophrenia: blunted, delayed, or normal?. Psychoneuroendocrinology.

[77] van Venrooij JA, Fluitman SB, Lijmer JG, Kavelaars A, Heijnen CJ, Westenberg HG (2012). Impaired Neuroendocrine and immune response to acute stress in medication-Naïve patients with a first episode of psychosis. Schizophr Bull.

[78] Jakovljevic M, Pivac N, Mihaljevic-Peles A, Mustapic M, Relja M, Ljubicic D (2007). The effects of olanzapine and fluphenazine on plasma cortisol, prolactin and muscle rigidity in schizophrenic patients: a double blind study. Prog Neuro-Psychopharmacol Biol Psychiatry.

[79] Popovic V, Doknic M, Maric N, Pekic S, Damjanovic A, Miljic D (2007). Changes in neuroendocrine and metabolic hormones induced by atypical antipsychotics in normal-weight patients with schizophrenia. Neuroendocrinology.

[80] Zhang XY, Zhou DF, Cao LY, GY W, Shen YC (2005). Cortisol and cytokines in chronic and treatment-resistant patients with schizophrenia: association with psychopathology and response to antipsychotics. Neuropsychopharmacology.

[81] Cohrs S, Röher C, Jordan W, Meier A, Huether G, Wuttke W (2006). The atypical antipsychotics olanzapine and quetiapine, but not haloperidol, reduce ACTH and cortisol secretion in healthy subjects. Psychopharmacology.

[82] Meltzer HY (1989). Clinical studies on the mechanism of action of clozapinee: the dopamine serotonin hypothesis of schizophrenia. Psychopharmacology.

[83] Benros ME, Nielsen PR, Nordentoft M, Eaton WW, Dalton SO, Mortensen PB (2011). Autoimmune diseases and severe infections as risk factors for schizophrenia: a 30-year population-based register study. Am J Psychiatry.

[84] Eaton WW, Byrne M, Ewald H, Mors O, Chen CY, Agerbo E (2006). Association of schizophrenia and autoimmune diseases: linkage of Danish national registers. Am J Psychiatry.

[85] Noto C, Ota VK, Gouvea ES, Rizzo LB, Spindola LM, Honda PH (2015). Effects of Risperidone on cytokine profile in drug-Naïve first-episode psychosis. Int J Neuropsychopharmacol.

[86] Stojanovic A, Martorell L, Montalvo I, Ortega L, Monseny R, Vilella E (2014). Increased serum interleukin-6 levels in early stages of psychosis: associations with at-risk mental states and the severity of psychotic symptoms. Psychoneuroendocrinology.

[87] Soderlund J, Schroder J, Nordin C, Samuelsson M, Walther-Jallow L, Karlsson H, et al. Activation of brain interleukin-1 beta in schizophrenia. Mol Psychiatry. 2009;14:1069–1071. 10.1038/mp.2009.52 PMC2848473 19920835

[88] Potvin S, Stip E, Sepehry AA, Gendron A, Bah R, Kouassi E (2008). Inflammatory cytokine alterations in schizophrenia: a systematic quantitative review. Biol Psychiatry.

[89] Dunjic-Kostic B, Jasovic-Gasic M, Ivkovic M, Radonjic NV, Pantovic M, Damjanovic A (2013). Serum levels of interleukin-6 and tumor necrosis factor-alpha in exacerbation and remission phase of schizophrenia. Psychiatr Danub.

[90] Chase KA, Cone JJ, Rosen C, Sharma RP (2016). The value of interleukin 6 as a peripheral diagnostic marker in schizophrenia. BMC Psychiatry.

[91] Andreassen OA, Harbo HF, Wang Y, Thompson WK, Schork AJ, Mattingsdal M, Zuber V, Bettella F, Ripke S, Kelsoe JR, Kendler KS, O'Donovan MC, Sklar P, LK ME, Desikan RS, Lie BA, Djurovic S, Dale AM, The Psychiatric Genomics Consortium (PGC) Bipolar Disorder and Schizophrenia Work Groups; The International Multiple Sclerosis Genetics Consortium (IMSGC) (2015). The psychiatric genomics consortium PGC bipolar disorder and schizophrenia work groups; the international multiple sclerosis genetics consortium IMSGC. Genetic pleiotropy between multiple sclerosis and schizophrenia but not bipolar disorder: differential involvement of immune-related gene loci. Mol Psychiatry.

[92] Network and Pathway Analysis Subgroup of the Psychiatric Genomics Consortium; International Inflammatory Bowel Disease Genetics Consortium (IIBDGC); International Inflammatory Bowel Disease Genetics Consortium IIBDGC (2015). Psychiatric genome-wide association study analyses implicate neuronal, immune and histone pathways. Nat Neurosci.

[93] Schizophrenia Working Group of the Psychiatric Genomics Consortium (2014). Biological insights from 108 schizophrenia-associated genetic loci. Nature.

[94] Stefansson H, Ophoff RA, Steinberg S, Andreassen OA, Cichon S, Rujescu D, Werge T, Pietiläinen OP, Mors O, Mortensen PB, Sigurdsson E, Gustafsson O, Nyegaard M, Tuulio-Henriksson A, Ingason A, Hansen T, Suvisaari J, Lonnqvist J, Paunio T, Børglum AD, Hartmann A, Fink-Jensen A, Nordentoft M, Hougaard D, Norgaard-Pedersen B, Böttcher Y, Olesen J, Breuer R, Möller HJ, Giegling I, Rasmussen HB, Timm S, Mattheisen M, Bitter I, Réthelyi JM, Magnusdottir BB, Sigmundsson T, Olason P, Masson G, Gulcher JR, Haraldsson M, Fossdal R, Thorgeirsson TE, Thorsteinsdottir U, Ruggeri M, Tosato S, Franke B, Strengman E, Kiemeney LA, Melle I, Djurovic S, Abramova L, Kaleda V, Sanjuan J, de Frutos R, Bramon E, Vassos E, Fraser G, Ettinger U, Picchioni M, Walker N, Toulopoulou T, Need AC, Ge D, Yoon JL, Shianna KV, Freimer NB, Cantor RM, Murray R, Kong A, Golimbet V, Carracedo A, Arango C, Costas J, Jönsson EG, Terenius L, Agartz I, Petursson H, Nöthen MM, Rietschel M, Matthews PM, Muglia P, Peltonen L, St Clair D, Goldstein DB, Stefansson K, Collier DA, Genetic Risk and Outcome in Psychosis (GROUP) (2009). Common variants conferring risk of schizophrenia. Nature.

[95] Chiappelli J, Shi Q, Kodi P, Savransky A, Kochunov P, Rowland LM (2016). Disrupted glucocorticoid-immune interactions during stress response in schizophrenia. Psychoneuroendocrinology.

[96] Handley R, Mondelli V, Zelaya F, Marques T, Taylor H, Reinders AA (2016). Effects of antipsychotics on cortisol, interleukin-6 and hippocampal perfusion in healthy volunteers. Schizophr Res.

[97] O'Connell KE, Thakore J, Dev KK (2014). Pro-inflammatory cytokine levels are raised in female schizophrenia patients treated with clozapine. Schizophr Res.

[98] Fonseka TM, Müller DJ, Kennedy SH (2016). Inflammatory cytokines and antipsychotic-induced weight gain: review and clinical implications. Mol Neuropsychiatry.

[99] Ng W, Kennar R, Uetrecht J (2014). Effect of Clozapinee and Olanzapine on Neutrophil kinetics: implications for drug-induced Agranulocytosis. Chem Res Toxicol.

[100] Pasquali R, Cantobelli S, Casimirri F, Capelli M, Bortoluzzi L, Flamia R (1993). The hypothalamic-pituitary-adrenal axis in obese women with different patterns of body fat distribution. J Clin Endocrinol Metab.

[101] Ohlson LO, Larsson B, Svardsudd K, Welin L, Eriksson H, Wilhelmsen L (1985). The influence of body fat distribution on the incidence of diabetes mellitus. 13.5 years of follow-up of the participants in the study of men born in 1913. Diabetes.

[102] Incollingo Rodriguez AC, Epel ES, White ML, Standen EC, Seckl JR, Tomiyama AJ (2015). Hypothalamic-pituitary-adrenal axis dysregulation and cortisol activity in obesity: a systematic review. Psychoneuroendocrinology.

[103] Baptista T, Mata A, Teneud L, De Quijada M, Han H-W, Hernandez L (1993). Effects of long term administration of clozapinee on body weight and food intake in rats. Pharmacol Biochem Behav.

[104] Ferno J, Ersland KM, Duus IH, González-García I, Fossan KO, Berge RK (2015). Olanzapine depot exposure in male rats: dose-dependent lipogenic effects without concomitant weight gain. Eur Neuropsychopharmacol.

[105] Taylor DM, Mc Askill R (2000). Atypical antipsychotics and weight gain-a systematic review. Acta Psychiatr Scand.

[106] Gressier F, Porcelli S, Calati R, Serretti A (2016). Pharmacogenetics of clozapinee response and induced weight gain: a comprehensive review and meta-analysis. Eur Neuropsychopharmacol.

[107] von Wilmsdorff M, Bouvier ML, Henning U, Schmitt A, Schneider-Axmann T, Gaebel W (2014). Sex-dependent metabolic alterations of rat liver after 12-week exposition to haloperidol or clozapine. Horm Metab Res.

[108] Kristiana I, Sharpe LJ, Catts VS, Lutze-Mann LH, Brown AJ (2010). Antipsychotic drugs upregulate lipogenic gene expression by disrupting intracellular trafficking of lipoprotein-derived cholesterol. Pharmacogenomics J.

[109] Lauressergues E, Staels B, Valeille K, Majd Z, Hum DW, Duriez P (2010). Antipsychotic drug action on SREBPs-related lipogenesis and cholesterogenesis in primary rat hepatocytes. Naunyn Schmiedeberg's Arch Pharmacol.

[110] Watanabe J, Suzuki Y, Sugai T, Fukui N, Ono S, Tsuneyama N (2012). The lipid profiles in Japanese patients with schizophrenia treated with antipsychotic agents. Gen Hosp Psychiatry.

