# Supplementary material for: Haloperidol affects bones while clozapine alters metabolic parameters - sex specific effects in rats perinatally treated with phencyclidine
Source: BMC Pharmacol Toxicol. 2017 Oct 11;18:65. doi: 10.1186/s40360-017-0171-4 (PMC5637335; doi:10.1186/s40360-017-0171-4)
Supplement: Supplementary file 1 — Fat content measured by DXA. (DOCX 20 kb) [file 40360_2017_171_MOESM1_ESM.docx]

**Additional file 1: Table S1.** Fat content measured by DXA.

| **FAT (g)** | | | | | | | |
| --- | --- | --- | --- | --- | --- | --- | --- |
|  | | **NaCl** | **PCP** | **NaCl-H** | **PCP-H** | **NaCl-C** | **PCP-C** |
| **Legs** male | PN  60 | 18.3±0.9 | 14.6±0.7^**^ | 15.5±0.2 | 13.6±1.3^***^ | 16.1±1 | 14.3±1.2^**^ |
|  | PN  98 | 18.5±1.23 | 23.3±2.2^**^ | 20.1±0.3 | 16.7±1.4^###^ | 22.4±0.9^*^ | 19.5±0.6^#^ |
| **Trunk** male | PN  60 | 45.9±3.8 | 35±1.6^*^ | 38.8±1.8 | 34.6±3.6^*^ | 42.4±3.9 | 36.5±2.9 |
|  | PN  98 | 62±4.7 | 64.8±3.6 | 58.1±2.6 | 56±2.8 | 68.7±2.1 | 67.1±3 |
| **Total** male | PN  60 | 69.6±4.8 | 53.9±2.2^*^ | 58.7±2.1 | 53.1±5.4^*^ | 63.2±4.9 | 55.3±4.4^*^ |
|  | PN  98 | 86.5±5.5 | 95.5±4.1 | 84.4±2.9 | 78.6±4^##^ | 99.1±2.5^*^ | 93.2±3.5 |
| **Legs** female | PN 60 | 15.3±0.9 | 10.7±0.4^**^ | 12.1±0.7^*^ | 11±1^***^ | 13.2±0.9 | 11.1±0.6^**^ |
|  | PN 98 | 15±0.9 | 13.3±0.7 | 15.6±0.6 | 14.6±0.9 | 16.3±0.7 | 14.9±0.5 |
| **Trunk** female | PN 60 | 45±2.9 | 33.7±1^**^ | 36±1.2^**^ | 32.3±2.6^***^ | 40.2±2.1 | 35.5±1.4^**^ |
|  | PN 98 | 62.3±2.4 | 49.6±1.9^***^ | 53.7±2.4^*^ | 47±2.3^***^ | 59.7±1.8 | 52.4±1.3^**^ |
| **Total** female | PN 60 | 66.7±3.4 | 48.2±1^***^ | 52.2±1.7^**^ | 48.2±3.9^***^ | 58.3±3 | 51±2^***^ |
|  | PN 98 | 83.9±3.3 | 68±2.4^***^ | 75.4±2.9 | 67.6±3.5^***^ | 83±2 | 73.7±1.7^*^ |

Effects of perinatal phencyclidine (PCP) treatment, haloperidol (H) and clozapine (C) on fat content on postnatal (PN) day 60 and PN 98 in male and female rats. Results are presented as mean values with standard error of the mean (SEM).

^*^p<0.05; ^**^p<0.01;^***^p<0.001 - comparing to control group

^#^p<0.05; ^##^p<0.01; ^###^p<0.001– comparing to PCP group
